# Epidemiology of Ocular Thelaziosis in Domestic Dogs in Beijing

**DOI:** 10.3390/pathogens13020166

**Published:** 2024-02-12

**Authors:** Zichen Liu, Chang Yu, Xiaoli Tan, Ni Chen, Yipeng Jin

**Affiliations:** Department of Clinical Veterinary Medicine, College of Veterinary Medicine, China Agricultural University, No. 2 Yuanmingyuan West Rd, Haidian District, Beijing 100193, China; lzc94@126.com (Z.L.); yuchangandy@gmail.com (C.Y.); Yuchen425@sina.com (X.T.); fuyingsiren@yeah.net (N.C.)

**Keywords:** thelaziosis, domestic dogs, vector-borne zoonosis, epidemiology, risk factor

## Abstract

*Thelazia callipaeda* is a zoonotic parasitic nematode that lives in the ocular conjunctival sac of domestic and wild carnivores, lagomorphs, and humans, with *Phortica* spp. as its intermediate host. At present, the important role that domestic dogs play in thelaziosis has been studied in many countries. However, Beijing, which is the first city in China to experience human thelaziosis, has not yet conducted a comprehensive epidemiological analysis of the disease. In this study, we analyzed risk factors (region, season, age, sex, breed, size, living environment, diet, country park travel history, immunization history, anthelmintic treatment history, and ocular clinical symptoms) associated with the prevalence of thelaziosis in domestic dogs in Beijing. The overall prevalence of *T. callipaeda* in the study area was 3.17% (102/3215 domestic dogs; 95% CI 2.57–3.78%). The results of the risk factor analysis showed that thelaziosis in domestic dogs from Beijing was significantly correlated with regional distribution, seasonal distribution, country park travel history, and anthelmintic treatment history (*p* < 0.05). In summer and autumn, domestic dogs living in mountainous areas, with a history of country park travel and without deworming were 4.164, 2.382, and 1.438 times more infected with *T. callipaeda* than those living in plain areas without a history of country park travel and with a history of deworming (OR = 4.164, OR = 2.382, OR = 1.438, respectively). *T. callipaeda*-infected domestic dogs did not always show any ocular clinical symptoms, while symptomatic domestic dogs were mainly characterized by moderate symptoms. The results indicate that in summer and autumn, preventive anthelmintic treatment should be strengthened for domestic dogs with a country park travel history or those living in mountain areas. At the same time, we should be vigilant about taking domestic dogs to play in country parks or mountainous areas during summer and autumn because this may pose a potential risk of the owner being infected with *T. callipaeda*.

## 1. Introduction

*Thelazia callipaeda* is a zoonotic parasitic nematode that lives in the ocular conjunctival sac of domestic and wild carnivores and lagomorphs, resulting in thelaziosis [1]. It uses *Phortica* spp. (fruit flies) as its intermediate host and transmission vector [2]. Because thelaziosis is distributed in many areas of Southeast Asia, it is also known as the oriental eyeworm [1]. The damage caused to the ocular area by *T. callipaeda* varies. Some infected dogs display foreign body sensations and increased ocular secretion, resulting in conjunctival edema, congestion, corneal ulcers, and even secondary glaucoma [3]. Severe cases can also cause blindness [1].

In 1917, human thelaziosis was reported for the first time in Beijing and Fujian, China [4,5]. In Europe, Italy has the largest number of cases of animal thelaziosis and is the first country to report the infection [1,6]. In the past 30 years, the number of cases of animal thelaziosis in Europe has gradually increased, including countries such as France, Spain, Belgium, Switzerland, Portugal, Romania, the United Kingdom, Greece, Hungary, Austria, and Germany [7,8,9,10,11,12,13,14,15,16,17]. From 1917 to 2020, a total of 658 cases of human thelaziosis were recorded in China [18]. The prevalence of thelaziosis is closely related to the age, sex, occupation, health condition, and density of the population within the epidemic area [18]. The data show that the prevalence of thelaziosis is highest in children aged 0–5 years old, which correlates strongly with their inability to avoid *Phortica* spp. The prevalence of thelaziosis is highest in outdoor occupations such as forest workers, which is related to the distribution density of *Phortica* spp. It is generally believed that infected domestic animals (dogs and cats) are the most important reservoir hosts of *T. callipaeda* [19], which directly threatens human health. Previously, cases of thelaziosis in dogs and humans were mainly reported in areas with poor economic and health conditions and large populations of domestic animals or wildlife [20,21]. In the villages around wildlife nature reserves in China, it was found that in 2019, the prevalence of domestic dog thelaziosis was as high as 84.62% (88/104), which was higher than that in 2016 (38.05%, 43/113), 2017 (53.92%, 55/102), and 2018 (56.25%, 63/112) [22]. Wildlife is also an important link in the transmission chain of *T. callipaeda*, especially in villages [9,23]. For example, a high-density distribution of red foxes in Europe often coincides with *Phortica* spp. during their active periods of the day; offspring red foxes’ habit of detaching and spreading from their original population also promotes the spread of *T. callipaeda* between regions [24,25]. Therefore, we should also pay attention to the role of wild carnivorous animals in introducing *T. callipaeda* into areas where there were no previous cases of thelaziosis, as well as their role in leading to the persistence and spread of an epidemic [24]. However, the overall impact of the diseases they cause in dogs and humans is not fully recognized in many respects, and 45% of the total human population of Europe, as well as their domestic and companion animals, are exposed to the risk of vector-borne zoonosis [26].

In order to investigate the prevalence of domestic dog thelaziosis in Beijing, this study collected clinical data on cases of domestic dog thelaziosis treated in the China Agricultural University Teaching Animal Hospital from 2018 to 2019, including region, season, age, sex, breed, size, living environment, diet, country park travel history, immunization history, anthelmintic treatment history, and ocular clinical symptoms. By organizing and analyzing the clinical data, we reveal the risk factors for the prevalence of domestic dog thelaziosis in Beijing, ultimately providing prevention and control strategies for local thelaziosis and maintaining local public health safety.

## 2. Materials and Methods

### 2.1. Sample Collection and Clinical Data Recording

Between 2018 and 2019, a total of 3215 domestic dogs were presented to the China Agricultural University Teaching Animal Hospital, located in Beijing (China), for an ocular health examination. Among them, 102 domestic dogs were infected with ocular nematodes. Infection was determined using a slit lamp under local anesthesia of the ocular region (0.5% proparacaine hydrochloride, Alcaine^®^, Alcon, Puurs, Belgium). These nematodes were removed and placed in a sterile collection tube containing 95% alcohol for subsequent identification. A total of 1277 nematodes were collected. We recorded the clinical data of each domestic dog, including region, season, age, sex, breed, size, living environment, diet, country park travel history (yes or no), immunization history (yes or no), anthelmintic treatment history (yes or no), and ocular clinical symptoms.

### 2.2. Morphological Observations

The nematodes collected from the eyes of each domestic dog were examined under an optical microscope combined with a camera (Leica, Wetzlar, Germany) and identified based on their morphology by using taxonomic keys [27].

### 2.3. Molecular Biology Confirmation

We extracted genomic DNA from nematodes collected from the conjunctival sacs of each domestic dog using HiPure Tissue & Blood DNA Kits (Magen, Guangzhou, China). A total of 102 samples were extracted. A partial sequence of the mitochondrial cytochrome c oxidase subunit 1 gene (*cox*1; 689 base pairs) was amplified using PCR [28]. Amplicons were purified by a HiPure Gel Pure Micro Kit (Magen, Guangzhou, China) and sequenced in an ABI3730XL with a BigDyeTr v3.1 Cycle Seq Kit (Applied Biosystems, Wakefield, RI, USA) [22]. Amplicon sequences were determined in both directions and genetic analyses were performed using available sequences of related nematodes from the GenBank and the Global Initiative on Sharing all Influenza Data (GISAID) database (https://www.gisaid.org, accessed on 24 January 2023).

### 2.4. Statistical Analysis

The clinical data of each domestic dog were entered into Microsoft Excel for classification and sorting, and the prevalence of different groups was calculated. The statistical analysis software SPSS Statistics v20 (IBM, New York, NY, USA) was adopted for statistics and data processing. The Chi-square test was used to compare and analyze the correlation between the prevalence of domestic dog thelaziosis and the risk factors (region, season, age, sex, breed, size, living environment, diet, country park travel history, immunization history, and anthelmintic treatment history) at a significance level of 5%. Associations between explanatory variables and the outcome were analyzed by logistic regression models [29]. The results were expressed as odds ratios (OR) with 95% confidence intervals (CI).

## 3. Results

### 3.1. Overall Prevalence of Thelaziosis in Domestic Dogs

**Figure 1:**
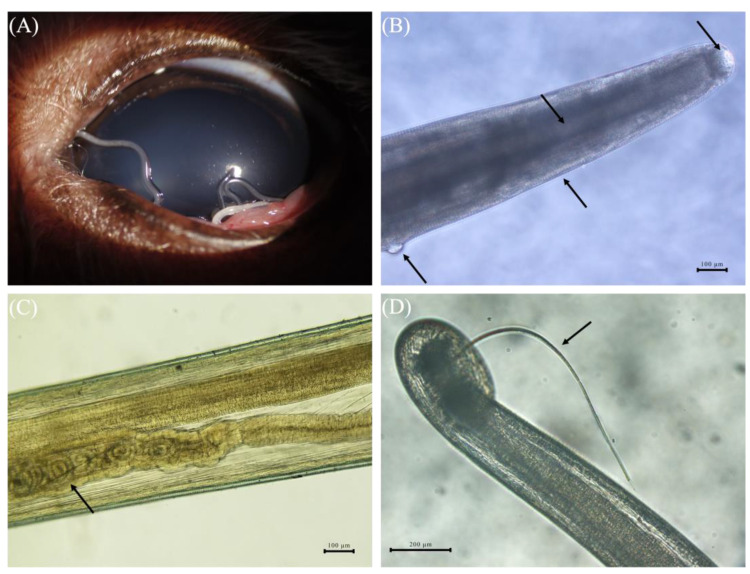
(**A**) *T. callipaeda* in the ocular conjunctival sac of a domestic dog. (**B**) Image of *T. callipaeda* showing the buccal capsule, pharynx, esophagus, cuticle striations, and vulva at the anterior end of a *T. callipaeda* female (arrow). (**C**) Larvae in a *T. callipaeda* female uterus (arrow). (**D**) The spicule at the posterior end of a *T. callipaeda* male (arrow).

As determined through slit-lamp observation, the nematode adults were slender, milky white, and cylindrical (Figure 1A). The buccal capsule, pharynx, esophagus, and cuticle striations were visible under the optical microscope (Figure 1B). Adult female nematodes possess a vulva at the anterior end (Figure 1B), as well as larvae and oval-shaped eggs in the mid-section of the uterus (Figure 1C). Adult male nematodes possess spicules that bend towards the ventral side at the posterior end (Figure 1D). A total of 1277 nematodes from these 102 domestic dogs were identified as *T. callipaeda* from their morphological characteristics. Out of the 1277 nematodes, 842 were morphologically identified as female and 435 as male *T. callipaeda*.

The morphological identification was confirmed by molecular analysis. The homology rate of nucleic acid samples from 102 domestic dogs was 100%. Through blast analysis, a representative sequence (GenBank accession no. MN719910) obtained from all the nematodes examined showed 98.79% nuclear similarity compared to the *T. callipaeda* sequence obtained from Chinese dogs (GenBank access no. MT040341).

The average number of *T. callipaeda* carried by each domestic dog was 7.80 ± 8.51 in the oculus sinister and 8.00 ± 9.08 in the oculus dexter. The overall prevalence of *T. callipaeda* was 3.17% (102/3215; 95% CI 2.57–3.78%) (Table 1).

### 3.2. Effect of Regional Distribution on Prevalence

**Figure 2:**
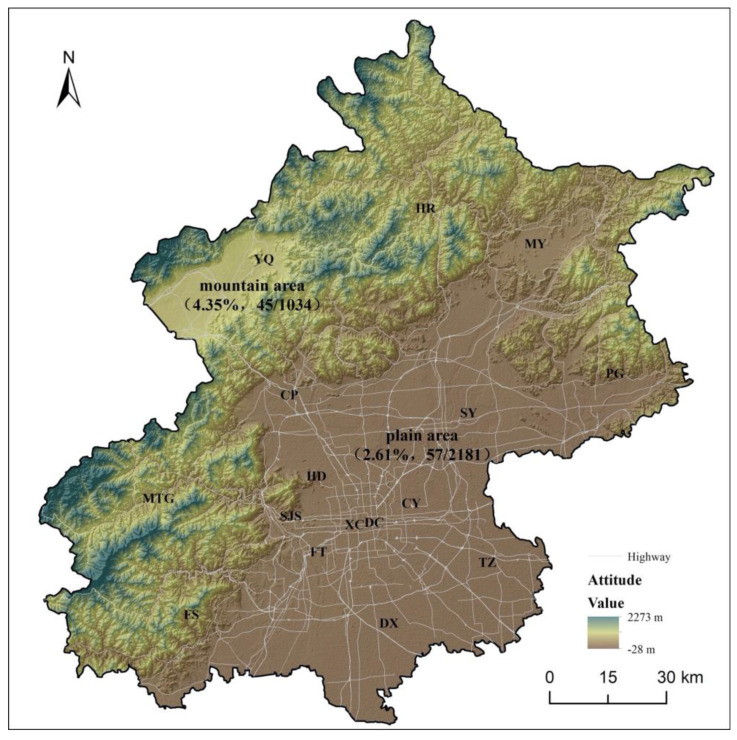
Prevalence and geographical location of domestic dog thelaziosis in mountainous areas and plain areas of Beijing from 2018 to 2019. DC: Dongcheng District, XC: Xicheng District, CY: Chaoyang District, HD: Haidian District, FT: Fengtai District, SJS: Shijingshan District, CP: Changping District, DX: Daxing District, FS: Fangshan District, MTG: Mentougou District, MY: Miyun District, PG: Pinggu District, SY: Shunyi District, TZ: TongZhou District, YQ: Yanqing District, and HR: Huairou District. Maps were created using ArcGIS 10.8.

Among the 3215 domestic dogs that underwent an ocular health examination in Beijing, 2181 domestic dogs came from plain areas and 1034 domestic dogs came from mountainous areas. Among the 2181 domestic dogs from plain areas, 57 cases were infected with *T. callipaeda*, with a prevalence of 2.61% (Figure 2). Among the 1034 domestic dogs from mountainous areas, 45 cases were infected with *T. callipaeda*, with a prevalence of 4.35% (Figure 2). The prevalence of thelaziosis in domestic dogs from mountainous areas was significantly higher than that from plain areas (χ^2^ = 6.902, *df* = 1, *p*-value = 0.009) (Table 1). Regional distribution had a significant effect on thelaziosis in domestic dogs.

### 3.3. Effect of Seasonal Distribution on Prevalence

The numbers of domestic dogs who underwent an ocular health examination in spring, summer, autumn, and winter were 780, 936, 827, and 672, respectively, for which the numbers of thelaziosis cases were 15, 40, 35, and 12, respectively. The prevalence of different seasonal distributions was 1.92% (15/780), 4.27% (40/936), 4.23% (35/827), and 1.79% (12/672), respectively. According to the Chi-square test (χ^2^ = 14.887, *df* = 3, *p*-value = 0.002), seasonal distribution had a significant effect on thelaziosis infection in domestic dogs (*p* < 0.05) (Table 1). The prevalence in summer (June to August) and autumn (September to November) was significantly higher than that in spring (March to May) and winter (December to February), but there was no significant difference between spring and winter or between summer and autumn.

### 3.4. Effect of Country Park Travel History on Prevalence

Among the 3215 domestic dogs who underwent an ocular health examination, 309 cases had a country park travel history and 2906 cases did not, for which the numbers of thelaziosis cases were 16 and 86, respectively. The prevalence of thelaziosis in dogs with different travel histories was 5.18% (16/309) and 2.96% (86/2906), respectively. According to the Chi-square test (χ^2^ = 4.475, *df* = 1, *p*-value = 0.034), the prevalence of thelaziosis in domestic dogs with a country park travel history was significantly higher than that in domestic dogs who had not traveled (*p* < 0.05) (Table 1).

### 3.5. Effect of Individual Factors on Prevalence

#### 3.5.1. Age

In this study, 102 cases of thelaziosis were identified in adult domestic dogs over 1 year old, aged between 1 and 15 years old. Therefore, puppies under 1 year old were excluded among the 3215 domestic dogs that underwent an ocular health examination, and 2768 adult domestic dogs’ clinical data were retained for statistical analysis. The numbers of domestic dogs who underwent an ocular health examination in the young (≤7 years old) and older (>7 years old) groups were 1592 and 1176, respectively, of which 66 and 36 were identified as thelaziosis cases, respectively. The prevalence of thelaziosis in the different age groups was 4.15% (66/1592) and 3.06% (36/1176), respectively. According to the Chi-square test (χ^2^ = 2.241, *df* = 1, *p*-value = 0.134), the age of domestic dogs had no significant effect on the prevalence of thelaziosis (*p* > 0.05) (Table 1).

#### 3.5.2. Sex

Among the 3215 domestic dogs who underwent an ocular health examination, 1821 were male and 1394 were female, for which the numbers of thelaziosis cases were 59 and 43, respectively. The prevalence of thelaziosis in the different sexes was 3.24% (59/1821) and 3.08% (43/1394), respectively. According to the Chi-square test (χ^2^ = 0.062, *df* = 1, *p*-value = 0.803), sex had no significant effect on the prevalence of thelaziosis (*p* > 0.05) (Table 1).

#### 3.5.3. Breed

The domestic dogs who underwent an ocular health examination collected in this study included 2753 purebred domestic dogs and 462 hybrid domestic dogs, for which the numbers of thelaziosis cases were 88 and 14, respectively. The prevalence of thelaziosis in the different breeds was 3.20% (88/2753) and 3.03% (14/462), respectively. According to the results of the Chi-square test (χ^2^ = 0.036, *df* = 1, *p*-value = 0.850), the breed had no significant effect on the prevalence of thelaziosis (*p* > 0.05) (Table 1).

#### 3.5.4. Size

Overall, 659, 836, 1479, and 241 domestic dogs who underwent an ocular health examination were small (<10 kg, <40 cm tall), medium (10–25 kg, 40–60 cm tall), large (26–45 kg, 61–70 cm tall), and giant domestic dogs (>45 kg, >70 cm tall), respectively, for which the numbers of thelaziosis cases were 25, 27, 41, and 9, respectively. The prevalence of thelaziosis in the different sizes of dogs was 3.79% (25/659), 3.23% (27/836), 2.77% (41/1479), and 3.73% (9/241), respectively. According to the results of the Chi-square test (χ^2^ = 1.856, *df* = 3, *p*-value = 0.603), size had no significant effect on the prevalence of thelaziosis (*p* > 0.05) (Table 1).

### 3.6. Effect of Feeding and Management Factors on Prevalence

#### 3.6.1. Living Environment

Overall, 2289, 416, and 510 domestic dogs came from a house, a kennel, or a free-range living environment, respectively, for which the numbers of thelaziosis cases were 67, 12, and 23, respectively. The prevalence of thelaziosis in the different living environments was 2.93% (67/2289), 2.88% (12/416), and 4.51% (23/510), respectively. According to the results of the Chi-square test (χ^2^ = 3.530, *df* = 2, *p*-value = 0.171), the living environment had no significant effect on the prevalence of thelaziosis (*p* > 0.05) (Table 1).

#### 3.6.2. Diet

According to the different types of food fed daily, the cases collected in this study were divided into two categories. Overall, 2607 domestic dogs ate commercial dog food, and 608 domestic dogs ate self-made food, for which the numbers of thelaziosis cases were 80 and 22, respectively. The prevalence of thelaziosis in dogs with different diets was 3.07% (80/2607) and 3.62% (22/608), respectively. According to the analysis results of the Chi-square test (χ^2^ = 0.485, *df* = 1, *p*-value = 0.486), the type of diet had no significant effect on the prevalence of thelaziosis (*p* > 0.05) (Table 1).

#### 3.6.3. Immunization History

A total of 2169 and 1046 domestic dogs in this study had been completely and incompletely immunized (within 1 year), respectively, for which the numbers of thelaziosis cases were 65 and 37, respectively. The prevalence of thelaziosis in dogs with and without complete immunization was 3.00% (65/2169) and 3.54% (37/1046), respectively. According to the analysis results of the Chi-square test (χ^2^ = 0.671, *df* = 1, *p*-value = 0.413), the completeness of immunization history had no significant effect on the prevalence of thelaziosis (*p* > 0.05) (Table 1).

#### 3.6.4. Anthelmintic Treatment History

The numbers of domestic dogs treated and not treated with an anthelmintic in the past 3 months were 2138 and 1077, respectively, for which the numbers of thelaziosis cases were 56 and 46, respectively. The prevalence of thelaziosis in dogs with and without an anthelmintic was 2.62% (56/2138) and 4.27% (46/1077), respectively. According to the Chi-square test (χ^2^ = 6.362, *df* = 1, *p*-value = 0.012), the prevalence of thelaziosis in domestic dogs without anthelmintic treatment was significantly higher than domestic dogs with anthelmintic treatment (*p* < 0.05) (Table 1).

### 3.7. Multivariate Analysis of Risk Factors Influencing the Prevalence of Domestic Dog Thelaziosis in Summer and Autumn

In summer and autumn, domestic dogs with thelaziosis were used as the dependent variable (No = 0 and Yes = 1), and the history of the region, country park travel history, and anthelmintic treatment history were used as independent variables. Logistic regression analysis showed that region, country park travel history, and anthelmintic treatment history were independent risk factors for the prevalence of domestic dog thelaziosis in summer and autumn (*p* < 0.05). In summer and autumn, domestic dogs living in mountainous areas, with a history of country parks travel and without deworming were 4.164, 2.382, and 1.438 times more infected with *T. callipaeda* than those living in plain areas without a history of country parks travel and with a history of deworming (OR = 4.164, OR = 2.382, and OR = 1.438, respectively) (Table 2).

### 3.8. Effect of T. callipaeda Carrying Capacity on Ocular Clinical Symptoms

A total of 111 infected eyes carried 1–10 *T. callipaeda*, 35 infected eyes carried 11–20 *T. callipaeda*, and 18 infected eyes carried over 20 *T. callipaeda*. It is worth noting that 11 *T. callipaeda*-infected eyes did not show any clinical symptoms, with 4 infected eyes carrying over 10 *T. callipaeda*. The results also found that the *T. callipaeda*-infected eyes with moderate symptoms (increased ocular secretion and purulent secretion with conjunctivitis) accounted for the largest proportion (Table 3).

## 4. Discussion

Further exploration of the relationship between prevalence and various risk factors showed that the epidemiological characteristics of thelaziosis were significantly correlated with regional distribution, seasonal distribution, country park travel history, and anthelmintic treatment history. However, they were not significantly correlated with individual factors (age, sex, breed, and size), living environment, diet, or immunization history. Multivariate analysis indicated that regional distribution, country park travel history, and anthelmintic treatment history were associated with an increased risk of domestic dog thelaziosis in summer and autumn.

Due to a relative lack of epidemiological survey data for domestic dog thelaziosis cases in China, compared with survey results from abroad, the total prevalence obtained in this test was low. Although it was similar to the prevalence (3.8%) and carrying capacity (8.08 ± 9.49) of pet dogs in Portugal [30], it was different from the prevalence (26.1%, 33.1%, and 68.0%) of dogs in Spain [31]. The variance was large, and the main reason may be the differences in sampling areas and subjects. The sampling site of this test was Beijing, and the sampling objects were mainly domestic dogs. The sampling area of dogs in Spain [31] was in the same latitude range as China, but there were many wildlife and fruit plantations in this area. In addition, the collection subjects with the highest prevalence (68.0%) were hounds with a high prevalence risk.

According to European survey data, in areas with high dog populations, there are more cases involving hosts such as cats and foxes [11,30]. In European countries where they are not endemic, the geographical distribution of thelaziosis is attributed to the dispersion of several wildlife species, including wild carnivores (such as the red fox, wolf, and badger) and rabbits [12]. According to a previous study, in Korea, the prevalence of thelaziosis in military dogs is as high as 33.5%, 12.4 times that of farm dogs, mainly because military dogs are raised in mountainous areas [32]. Research results support not only the existence of a mountainous area life cycle of *T. callipaeda* but also indicate that it is maintained mainly by foxes and secondarily by a large number of wildlife species [33]. In 2019, giant pandas, wild boars, leopard cats, and black bears were found to be newly infected with *T. callipaeda* in the Qinling Mountains of China. The prevalence of domestic dog thelaziosis in the surrounding villages also showed an increasing trend year by year, and the density of *Phortica* spp. in the mountainous area increased sharply; these results indicate that there is a transmission cycle of *T. callipaeda* among wildlife in the mountainous areas of China [22]. The results of our study and previous studies suggest that domestic dogs frequently exposed to mountainous areas or country parks are more likely to come into contact with *T. callipaeda*-infected wildlife and *Phortica* spp., resulting in a relatively higher prevalence of thelaziosis.

The results showed that seasonal distribution has a significant effect on thelaziosis infection in domestic dogs. The prevalence in summer and autumn was significantly higher than in spring and winter. This result is related to the reproductive law of *Phortica* spp., whose reproduction, density, and biological activity vary seasonally according to climatic conditions [2,22]. Therefore, thelaziosis occurrence may be seasonal. According to a study on *Phortica* spp. in Spain, the number of *Phortica* spp. captured was largest when the average daily temperature was 24.5–26.0 °C, and no *Phortica* spp. were captured when the daily average temperature was 8.8 °C; the density and number of *Phortica* spp. increased with warmer temperatures, and the proportion of male *Phortica* spp. (79.65%) was significantly higher than female *Phortica* spp. (20.35%) [31]. Generally, only male *Phortica* spp. feed on animals and human ocular secretions [1]. Therefore, the number of male *Phortica* spp. increases significantly with warmer summer temperatures. The transmission capacity also peaks during this time, and thus, the prevalence is at its highest in summer and autumn. In addition, the study data also showed that the proportion of male *Phortica* spp. is positively correlated with the temperature at the time of capture, and at lower temperatures, the proportion of female *Phortica* spp. will be higher [34]. As mature female *T. callipaeda* continuously releases larvae (L1) during the active period to maintain their reproductive cycle, infectious *Phortica* spp. from the previous year can move again in early spring after overwintering [35]. Therefore, there is still a risk of infection in spring and winter.

In this study, the prevalence of thelaziosis in domestic dogs without deworming was significantly higher than in domestic dogs with deworming. Anthelmintics can have an effect on thelaziosis prevention. Because the animal owners were unsure about the compounds of the anthelmintic drugs purchased, there was no analysis of the relationship between the compounds of the anthelmintic drugs used on domestic dogs and the prevalence of thelaziosis. Many studies have shown that the application of moxidectin, imidacloprid, and milbemycin oxime was highly effective in the treatment of canine thelaziosis caused by *T. callipaeda* [25,36,37]. These compounds are also common in most anthelmintic drugs for dogs that are currently available in China. In the future, more clinical data on anthelmintic drugs (such as active compounds and frequency of use) should be collected for further study.

According to the analysis, the number of monocular *T. callipaeda* and ocular clinical symptoms from 102 domestic dog thelaziosis cases were tallied and classified. Cases with moderate symptoms (increased ocular secretion and purulent secretion with conjunctivitis) accounted for the largest proportion, although there were domestic dogs with *T. callipaeda* that did not show any ocular clinical symptoms (Table 3). In general, because the movement of *T. callipaeda* directly damages the conjunctival sac and other parts, it is reasonable to deduce that ocular clinical symptoms will increase with the number of monocular *T. callipaeda*. However, our results do not support this assumption. According to a previous study, the severity of ocular clinical symptoms is not related to the number of infected nematodes [38], which concurs with the results of this test. Other study data show that the prevalence of thelaziosis in dogs with ocular clinical symptoms was significantly higher than in asymptomatic dogs [30]. However, it is also emphasized that in the early stages of infection, animals usually do not exhibit obvious ocular clinical symptoms; therefore, they may be missed by owners and veterinarians.

## 5. Conclusions

Thelaziosis infection in domestic dogs from Beijing was significantly correlated with regional distribution, seasonal distribution, country park travel history, and anthelmintic treatment history. In addition, the regional distribution, country park travel history, and anthelmintic treatment history were associated with an increased risk of domestic dog thelaziosis in summer and autumn. This suggests that if a domestic dog has a history of country park travel or lives in a mountainous area, whether there are ocular clinical symptoms or not, regular internal and external anthelmintics should be administered according to the procedure. Furthermore, we should be vigilant about taking domestic dogs to play in country parks or mountainous areas during summer and autumn because this may pose a potential risk of the owner being infected with *T. callipaeda*. The owner should pay special attention to ocular hygiene and guard against the possibility of zoonotic diseases.

## Figures and Tables

**Table 1:** Risk factors and prevalence of thelaziosis infection in domestic dogs from Beijing in 2018–2019.

Risk Factors		Frequency	Prevalence	95% CI	χ^2^ (*df*)	*p*-Value
Region	Plain area	57/2181	2.61%	1.94–3.28%	6.902 (1)	0.009
	Mountainous area	45/1034	4.35%	3.11–5.60%		
Season	Spring	15/780	1.92%	0.96–2.89%	14.887 (3)	0.002
	Summer	40/936	4.27%	2.98–5.57%		
	Autumn	35/827	4.23%	2.86–5.60%		
	Winter	12/672	1.79%	0.78–2.79%		
Age	≤7Y	66/1592	4.15%	3.17–5.12%	2.241 (1)	0.134
	>7Y	36/1176	3.06%	2.08–4.05%		
Sex	Male	59/1821	3.24%	2.43–4.05%	0.062 (1)	0.803
	Female	43/1394	3.08%	2.18–3.99%		
Breed	Pure breed	88/2753	3.20%	2.54–3.85%	0.036 (1)	0.850
	Half breed	14/462	3.03%	1.47–4.59%		
Size	Small	25/659	3.79%	2.34–5.25%	1.856 (3)	0.603
	Medium	27/836	3.23%	2.03–4.43%		
	Large	41/1479	2.77%	1.94–3.61%		
	Giant	9/241	3.73%	1.34–6.13%		
Living environment	House	67/2289	2.93%	2.24–3.62%	3.530 (2)	0.171
	Kennel	12/416	2.88%	1.28–4.49%		
	Free range	23/510	4.51%	2.71–6.31%		
Diet	Commercial food	80/2607	3.07%	2.41–3.73%	0.485 (1)	0.486
	Self-made food	22/608	3.62%	2.13–5.10%		
Country park travel history	Yes	16/309	5.18%	2.71–7.65%	4.475 (1)	0.034
	No	86/2906	2.96%	2.34–3.58%		
Immunization history	Yes	65/2169	3.00%	2.28–3.71%	0.671 (1)	0.413
	No	37/1046	3.54%	2.42–4.66%		
Anthelmintic treatment history	Yes	56/2138	2.62%	1.94–3.30%	6.362 (1)	0.012
	No	46/1077	4.27%	3.06–5.48%		
Total		102/3215	3.17%	2.57–3.78%	-	-

Frequency: thelaziosis cases/ophthalmic cases, CI: confidence interval, *df*: degree of freedom, spring: March to May, summer: June to August, autumn: September to November, and winter: December to February.

**Table 2:** Analysis of risk factors influencing the prevalence of domestic dog thelaziosis in summer and autumn.

Risk Factors	OR	95% CI	*p*-Value
Region	4.164	1.023–19.437	0.023
Country park travel history	2.382	1.039–28.831	0.015
Anthelmintic treatment history	1.438	1.009–2.243	0.022

OR: Odds Ratio and CI: confidence interval.

**Table 3:** Correlation between ocular clinical symptoms and *Thelazia callipaeda* carrying capacity.

Ocular Clinical Symptoms	Number of Monocular *T. callipaeda*		
	1–10	11–20	>20
	Prevalence		
Asymptomatic	6.31% (7/111)	8.57% (3/35)	5.56% (1/18)
Mild	36.94% (41/111)	14.29% (5/35)	5.56% (1/18)
Moderate	47.75% (53/111)	62.86% (22/35)	66.67% (12/18)
Severe	9.00% (10/111)	14.29% (5/35)	22.22% (4/18)

Mild symptoms: increased ocular secretion and purulent secretion, moderate symptoms: increased ocular secretion and purulent secretion with conjunctivitis, and severe symptoms: keratitis and corneal ulcer.

## Data Availability

The datasets supporting the conclusions of this article are included within this article.
